# Interrater reliability of motor severity scales for hemifacial spasm

**DOI:** 10.1007/s00702-023-02667-w

**Published:** 2023-07-19

**Authors:** Ha Yeon Lee, Ingyun Park, Minnie P. Luu, Jerry Zhao, Jeanne P. Vu, Elizabeth Cisneros, Brian D. Berman, H. A. Jinnah, Han-Joon Kim, Catherine Y. Liu, Joel S. Perlmutter, Sarah Pirio Richardson, Anne Weissbach, Glenn T. Stebbins, David A. Peterson

**Affiliations:** 1 Institute for Neural Computation, University of California, San Diego, La Jolla, CA USA; 2 Department of Neurology, Virginia Commonwealth University, Richmond, VA USA; 3 Department of Neurology, Emory University School of Medicine, Atlanta, GA USA; 4 Department of Human Genetics, Emory University School of Medicine, Atlanta, GA USA; 5 Department of Neurology and Movement Disorders Centre, Seoul National University Hospital, Seoul, South Korea; 6 Viterbi Family Department of Ophthalmology, University of California, San Diego, La Jolla, USA; 7 Department of Ophthalmology, Rady Children’s Hospital, San Diego, CA USA; 8 Division of Oculofacial Plastic and Reconstructive Surgery, Shiley Eye Institute, University of California, San Diego, La Jolla, USA; 9 Department of Neurology, Washington University School of Medicine, St. Louis, MO USA; 10 Departments of Radiology, Neuroscience, Physical Therapy, and Occupational Therapy, Washington University School of Medicine, St. Louis, MO USA; 11 Department of Neurology, University of New Mexico Health Sciences Center, Albuquerque, NM USA; 12 Neurology Service, New Mexico Veterans Affairs Health Care System, Albuquerque, NM USA; 13 Institute of Systems Motor Science, University of Luebeck, Luebeck, Germany; 14 Institute of Neurogenetics, University of Luebeck, Luebeck, Germany; 15 Department of Neurological Sciences, Rush University Medical Center, Chicago, IL USA; 16 Computational Neurobiology Laboratory, Salk Institute for Biological Studies, 10010 N. Torrey Pines Rd, La Jolla, CA 92037 USA

**Keywords:** Severity rating scale, Hemifacial spasm, Interrater reliability

## Abstract

To compare the inter-rater reliability (IRR) of five clinical rating scales for video-based assessment of hemifacial spasm (HFS) motor severity. We evaluated the video recordings of 45 HFS participants recruited through the Dystonia Coalition. In Round 1, six clinicians with expertise in HFS assessed the participants’ motor severity with five scales used to measure motor severity of HFS: the Jankovic rating scale (JRS), Hemifacial Spasm Grading Scale (HSGS), Samsung Medical Center (SMC) grading system for severity of HFS spasms (Lee’s scale), clinical grading of spasm intensity (Chen’s scale), and a modified version of the Abnormal Involuntary Movement Scale (Tunc’s scale). In Round 2, clinicians rated the same cohort with simplified scale wording after consensus training. For each round, we evaluated the IRR using the intraclass correlation coefficient [ICC (2,1) single-rater, absolute-agreement, 2-way random model]. The scales exhibited IRR that ranged from “poor” to “moderate”; the mean ICCs were 0.41, 0.43, 0.47, 0.43, and 0.65 for the JRS, HSGS, Lee’s, Chen’s, and Tunc’s scales, respectively, for Round 1. In Round 2, the corresponding IRRs increased to 0.63, 0.60, 0.59, 0.53, and 0.71. In both rounds, Tunc’s scale exhibited the highest IRR. For clinical assessments of HFS motor severity based on video observations, we recommend using Tunc’s scale because of its comparative reliability and because clinicians interpret the scale easily without modifications or the need for consensus training.

## Introduction

Hemifacial spasm (HFS) is a chronic condition characterized by involuntary contractions of facial muscles occurring on one side of the face (Jamjoom et al. 1990), with a prevalence of about 10 per 100,000 individuals (Auger and Whisnant 1990; Nilsen et al. 2004). Approximately 90% of patients suffer from social isolation and depression (Rudzińska et al. 2012), which can hinder their career and social life (Costa et al. 2005). The main treatments for HFS are botulinum neurotoxin injections and microvascular decompression surgery of the facial nerve (Dannenbaum et al. 2008).

Determining the efficacy of HFS treatment relies on the assessment of the severity of motor manifestations, typically with clinical rating scales. Several scales for rating motor severity have been used for HFS (see Table 1). Some scales commonly used for rating HFS motor severity were initially designed for other movement disorders, involving general facial and orbicularis oculi spasms as seen with blepharospasm. Although the scales vary in terms of the specific motor phenomena they are meant to capture, they have all been used to assess overall motor severity. All of the scales are ordinal with small ranges (i.e., 1–3 or 0–4), which limits sensitivity to small differences or changes in severity (Wabbels and Roggenkämper 2012). There is no general agreement on a single standard rating scale for HFS (Wabbels and Roggenkämper 2012). The multitude of different rating scales used to measure HFS motor severity in past studies and the lack of standardization among them make it difficult to compare different trials (Wabbels and Roggenkämper 2012).

**Table 1:** Hemifacial spasm rating scales

Abbreviation	Full scale title	References
JRS	Jankovic rating scale	Jankovic (1987)
HSGS	The Hemifacial Spasm Grading Scale	Tambasco et al. (2019)
Lee’s scale	SMC grading system for HFS	Lee et al. (2012)
Chen’s scale	Clinical grading of spasm intensity	Chen (1996)
Tunc’s scale	Abnormal involuntary movement scale	Tunc (2008), Guy (1976)

Furthermore, all the scales are based on human judgment and are therefore susceptible to inter-rater variability (Wabbels and Roggenkämper 2012). Some of the individual scales have been assessed for their inter-rater reliability (IRR). The HSGS [ICC = 0.62–0.82 (Tambasco et al. 2019); Spearman’s rho = 0.96] (Osaki et al. 2020b), JRS (Spearman’s rho = 0.95) (Osaki et al. 2020b), and Lee’s scale (kappa = 0.86, CI 0.7941–0.9321) (Lee et al. 2012) have demonstrated what the authors of those studies characterize as good IRR. However, Chen’s and Tunc’s scales have not yet, to our knowledge, been evaluated for IRR when used for HFS.

Single studies comparing multiple HFS severity rating scales are sparse. Only two pairs of HFS scales have been directly compared for IRR: (1) the HSGS and Chen’s scale exhibited good correlations across individual raters and pre-/post-treatment (Spearman’s rho = 0.61–0.84) (Tambasco et al. 2019) and (2) the HSGS and JRS exhibited high correlations (Spearman’s rho = 0.92) (Osaki et al. 2020b). However, these separate studies cannot be combined, because they used different sets of patients and raters. To our knowledge, no study evaluated IRR across all these scales with a single set of patients and raters.

In this cross-sectional study, our primary objective was to evaluate and compare the IRR of five clinical rating scales that have been utilized for quantifying HFS motor severity. We used the same 6 raters and the same cohort of 45 participants across all scales. We used video-based assessments, because this ensured that each rater viewed the same manifestations under the same conditions. Video-based ratings also have become increasingly important for multisite/multi-rater trials, facilitate review and comparisons across multiple visits for individual participants, and permit remote, telemedicine-based studies that can incorporate more frequent at-home assessments. Clinical trials in movement disorders often involve one or more “consensus training” meetings among the multiple raters involved in the study to discuss how the scales are interpreted and should be applied in a fashion standardized across raters (Sadler et al. 2017). This type of rater training improves IRR (Müller and Wetzel 1998). Thus, our secondary objective was to determine to what extent a similar process of consensus training would improve the reliability of these HFS scales.

## Methods

We analyzed single standardized video recordings of 53 HFS patients previously enrolled in an observational study across ten tertiary research sites (Defazio et al. 2021) affiliated with the Dystonia Coalition (http://www.rarediseasesnetwork.org/dystonia) (Kilic-Berkmen et al. 2021). The protocols were approved by the Human Research Protection Office at Washington University School of Medicine and the University of California, San Diego (protocol 111255X). All patients provided informed consent prior to participation. The exclusion criteria were secondary blepharospasm and other co-existing medical conditions and surgical interventions that may confound assessments. All patients had their last botulinum neurotoxin injections performed at least 10 weeks prior, so that symptoms would be evident upon observation. Participant demographics are provided in Table 2. Information about age at onset, symptoms duration, left vs. right HFS, and specific muscles involved were not collected during original data acquisition.

**Table 2:** Patient demographics (*N* = 45)

Age at exam (years)	Range	41–86
	Mean	66.8
	SD	10.2
Gender	Female	24
	Male	21
Race	White	41
	Asian	2
	Black	1
	Other	1

Two annotators independently reviewed all participant videos, marking the beginning and end times of a passive “observation” period using Elan 4.9.4 (Charles et al. 2014; ELAN 2017). The observation period started when the proctor instructed the participant to blink normally looking at the camera and usually lasted for about 2 min. The overlap of the annotators’ time segments determined the precise observation period used for this study. Some rating scales include language implying that participants are asked to assume specific postures or do specific activities that activate (e.g., evoke or worsen) their symptoms. Although such a procedure was used in the Dystonia Coalition’s examination protocol, it was not administered consistently across participants. Thus, for this study, we used only the observation period in our analyses.

All participants were rated by 6 clinicians (five movement disorders neurologists and one neuro-ophthalmologist, hereafter referred to as raters) with expertise in HFS (BDB, HJK, CYL, JSP, SPR, and AW). Raters were instructed to watch the observation period to assess each participant. They rated overall HFS motor severity using five scales (Table 1): (1) the JRS, (2) HSGS, (3) the “Samsung Medical Center (SMC) grading system for HFS” (what we refer to as “Lee’s scale”, (4) the “Clinical grading of spasm intensity” (what we refer to as “Chen’s scale”), and (5) a modified usage of the Abnormal Involuntary Movement Scale commonly used to assess tardive dyskinesia [AIMS (Guy 1976); that incorporates only the AIMS item “Muscles of facial expression”; what we refer to as “Tunc’s scale”].

As many clinical trials in the movement disorders’ field involve consensus training, we sought to specifically assess whether consensus training improved the IRR of the HFS scales. Thus, we had the raters rate all the participants with the scales in two rounds with consensus training between the first and second rounds. In Round 1, participant videos were rated with no prior consensus training (Table 3).

**Table 3:** Scale score distributions (*N* = 270)

Scale	Statistic	Round 1	Round 2
JRS	Median	4	4
	Mean	3.5	3.5
	SD	2.5	2.5
	Range	0–8	0–8
HSGS	Median	6	6
	Mean	5.4	5.3
	SD	2.7	3.1
	Range	0–9	0–9
Lee	Median	2	2
	Mean	1.7	1.7
	SD	1.0	1.2
	Range	0–4	0–4
Chen	Median	2	3
	Mean	1.8	2.1
	SD	1.3	1.4
	Range	0–4	0–4
Tunc	Median	2	2
	Mean	1.7	1.8
	SD	1.2	1.3
	Range	0–4	0–4

After Round 1, it became evident that many aspects of the scales made their application to only video-based observation problematic. In each case, we identified the issues and addressed them through adaptations to the scales. Overall, this involved simplifying the language of the scales to facilitate their clear standardized application (see Appendix A). All scales except for Tunc’s scale had rating scale anchor words and phrases that were ambiguous and therefore susceptible to variable interpretation. For example, the JRS and Chen scales refer to “fluttering” without explicitly defining it. Collectively, the raters agreed on a definition of “fluttering” as blinks occurring in rapid succession, sometimes without a complete opening of the eye between blinks. Many anchors also depended on conditional actions, including various “activation” procedures, involving asking participants to assume specific postures or do specific activities that would modify—usually evoke or worsen—symptoms (e.g., “only with external stimuli”, “provoked by motor activation”, etc.). In some cases, these activation procedures were not defined in the scale instructions nor the scale’s original paper. In addition, some anchors would implicitly be best answered by direct participant input (e.g., “functionally disabling”, “incapacitating social activities”, “interference with vision”, etc.). In all these cases, ratings would differ depending on whether the raters interpreted them literally or figuratively (i.e., the “letter of the law” or the “spirit of the law”). Thus, these ambiguous phrases were omitted. The HSGS and Lee’s scale did not have a 0 option, leading the raters to disagree about how to rate participants with no symptoms observed during the video’s observation period, giving a score (1) according to the scale, (2) of “N/A”, or (3) of 0. Thus, for these two scales, we added a 0 option.

Once the wording of the scales was modified, the raters underwent a training session to review the changes and practice their application. We used Tunc’s scale to identify five participant videos to review, because it exhibited the highest IRR from Round 1 (see “Results”). These five participants were chosen to represent each of the five possible levels of severity in Tunc’s scale, and they were excluded from subsequent ratings and the overall analyses. During the training, the five participants were reviewed in random order of severity. The raters first independently evaluated each example participant and then collectively discussed their scores, resulting in a consensus on how to rate the participants for all five scales. In Round 2, the raters used the updated scales. The same videos were used in both rounds to eliminate the possibility that differences in ratings would be the result of differences in the video recordings.

We evaluated the IRR for each scale and each round using the Intraclass Correlation Coefficient (ICC). Out of the 53 participants, three were excluded, because they were re-diagnosed by participating raters as not having HFS (one had oromandibular dystonia, one had synkinesis from Bell’s palsy, and one had an unclear diagnosis). Five more were excluded, because they were used as examples during the consensus training. The subsequent 45 participants were used to calculate the ICC. Because we wanted our ICC estimates to generalize to randomly selected samples of both all possible patients in this population and to all possible raters, we used a single-rater, absolute-agreement, 2-way random model for both rounds [i.e., ICC(2,1)] (McGraw and Wong 1996). With this size cohort, we had 90% power to detect an ICC value as low as 0.2 (Bujang and Baharum 2017). To conservatively characterize each scale’s inter-rater reliability, we used the lower bound of the 95% confidence interval (Koo and Li 2016). The level of reliability is defined, such that values less than 0.5 are poor, between 0.5 and 0.75 are moderate, between 0.75 and 0.9 are good, and above 0.9 are excellent (Koo and Li 2016). To compare the IRR between scales and rounds, we sought to determine whether the ICCs’ 95% confidence intervals overlap, as has been used in prior studies with separate groups of trained and untrained raters evaluating the effect of rater training (Robertson et al. 2020).

## Results

**Fig. 1:**
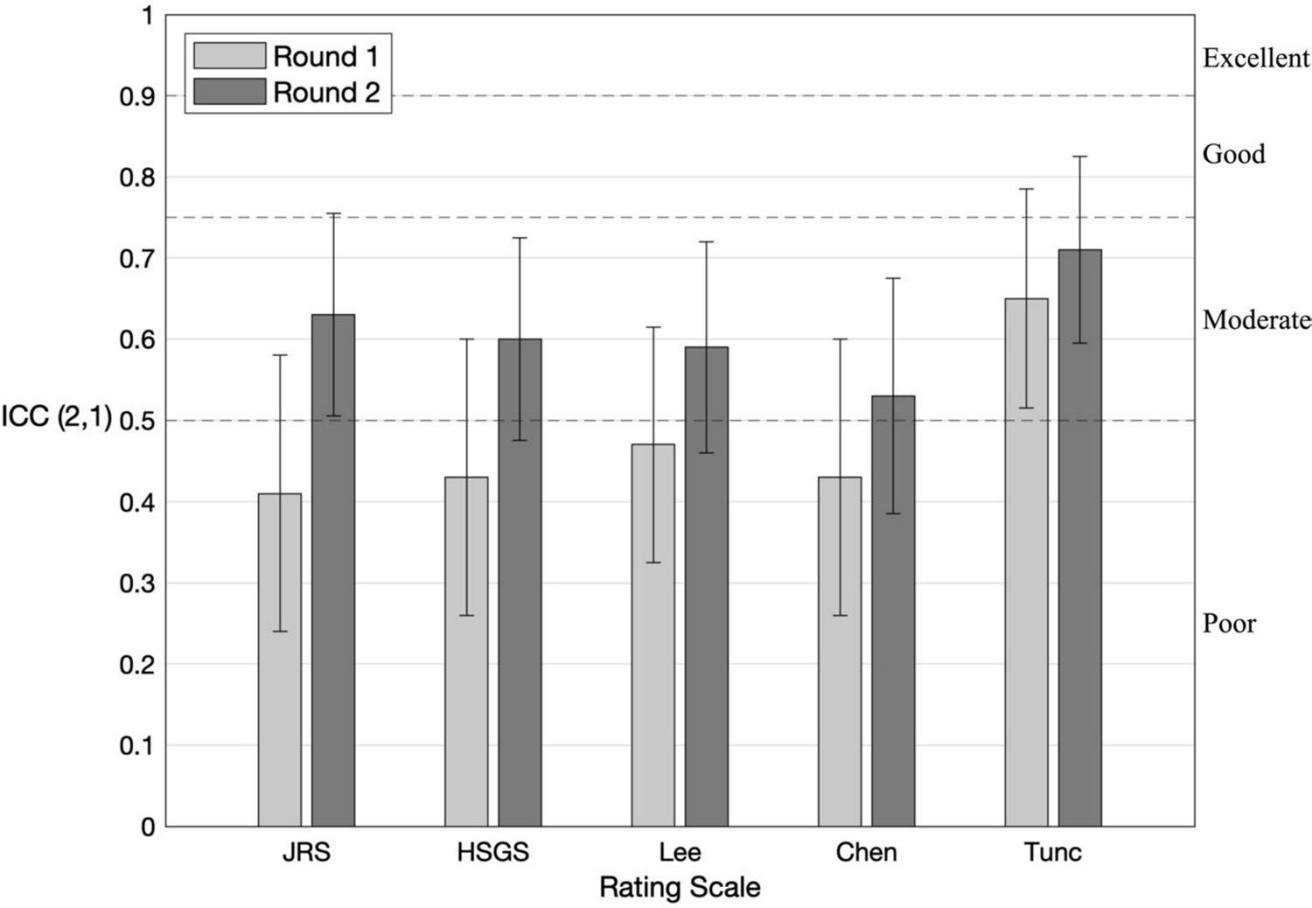
Interrater reliability for HFS rating scales. Intraclass Correlation Coefficient (ICC) values from Round 1 and Round 2. Whiskers indicate 95% confidence interval. Dashed lines delineate conventional thresholds for interpreting reliability associated with ICC values (poor < 0.5, moderate 0.5–0.75, good 0.75–0.9, and excellent > 0.9) (Koo and Li 2016). See Table 1 for scale abbreviations

Descriptive statistics of the ratings for all scales and both Rounds are provided in Table 3. In Round 1, the IRR was “poor” for the JRS, HSGS, Lee’s scale, and Chen’s scale and “moderate” for Tunc’s scale (mean ICC = 0.41, 0.43, 0.47, 0.43, 0.65, respectively; see Fig. 1).

There was an interval in the range of 138–184 days between raters completing their Round 1 and Round 2 ratings. In Round 2, the IRR was “moderate” for JRS, “poor” for HSGS, Lee’s scale, and Chen’s scale, and “moderate” for Tunc’s scale (mean ICC = 0.63, 0.60, 0.59, 0.53, 0.71 respectively; see Fig. 1). The mean ICC increased for all 5 scales in Round 2 compared to Round 1. However, for every scale, the 95% CI for the two rounds overlapped, implying no significant improvement of each of the five scales’ IRR with training.

Across both rounds, Tunc’s scale was the most reliable out of the five scales.

We conducted multiple post hoc analyses. In terms of the ICC measures of IRR, if the ratings were averaged across the six raters, their reliability [ICC (2,6) average-rater, absolute-agreement, 2-way random model] was 0.80, 0.82, 0.84, 0.82, and 0.92 in Round 1 and 0.91, 0.90, 0.90, 0.87, and 0.94 in Round 2 for JRS, HSGS, Lee’s, Chen’s, and Tunc’s scale, respectively. We also calculated several less systematic non-ICC measures of reliability (see Appendices for results): (1) how much raters differed in their ratings between the two rounds (Appendix B), (2) how much raters differed among themselves in how severely they rated patients on average (Appendix C), (3) the proportion of ratings for which the raters were within one point of each other (Appendix D), and (4) the distribution across patients of inter-rater variability (as measured by the median absolute deviation of ratings normalized by each scale range, Appendix E).

## Discussion

### Overall IRR results

We evaluated the inter-rater reliability (IRR) of five rating scales previously used to assess motor severity of HFS. We controlled for the influence of raters and participants using the same six raters, the same training process, and the same cohort of 45 participants for all scales. We also controlled for the different degrees of granularity among the scales using the Intraclass Correlation Coefficient to evaluate their IRR. All of the scales exhibited poor-to-moderate reliability. The result is not surprising, because most severity rating scales used in movement disorders suffer from suboptimal IRR (Fearon et al. 2019).

### Adapting the scales

After the first round of ratings, the raters identified aspects of most of the scales that made them difficult to use in our context of passive, observational assessments based on video recordings. This was probably because the scales were originally developed for use with live, interactive assessments of participants. To streamline the process for applying these scales to our observational, video-based assessments, we adapted the scales for the “consensus training” and their use in the second round of ratings. The overarching intent was to make clear how the scales should be interpreted and applied to videos in a standardized fashion across raters.

As detailed in the Methods, the scales were adapted to address: (1) language that was ambiguous and susceptible to variable interpretation, (2) the omission of a “0” score for rating no observable symptoms, (3) assessment that requires participant input and cannot be ascertained from observation alone, and (4) undefined activating procedures. The latter two reasons for adapting the scales depend not only on the rating scale, but also on the examination protocol. Across movement disorders, there has been a lot of effort devoted to developing and validating rating scales, but comparatively less effort devoted to precisely specifying standardized examination protocols. This discrepancy is particularly significant when patients are video recorded, and those recordings used for ratings. Yet video-based assessments offer several advantages over live, in-person assessments. They enable: (1) standardized “input data” for the rating process, (2) review of any given individual patient by multiple raters, and (3) convenient assessments over time and through telemedicine. Standardizing the rating scales as well as the examination protocols would enable the scales to generalize to other video-based studies and facilitate meta-analyses combining results across different studies.

### Effect of “consensus training”

Movement disorders’ clinical trials often involve one or more “consensus training” meetings among raters to achieve consensus about how the scales should be interpreted and applied (Sadler et al. 2017). In a study using the Barry-Albright Dystonia Scale and video recordings of participants with dystonia to assess their motor severity, the scale’s IRR increased after the clinicians underwent rater training (Barry et al. 1999). Thus, we sought to determine whether and to what extent rater training would improve the IRR for HFS scales. Interestingly, although we found a trend toward improved IRR across all of the scales, it was not a significant improvement for any individual scale. Relatedly, raters exhibited systematic differences in how severely they rated patients even averaged across the whole cohort (Appendix C). These likely reflect intrinsic rater biases in motor severity assessments, because although they were partly attenuated by after consensus training, the same pattern of inter-rater differences was still evident.

The time intervals between Round 1 and Round 2 differed among the raters, potentially differentially impacting the effectiveness of the consensus training. However because the range of that interval was 138–184 days, given the amount and variety of these clinicians’ workloads, we expected there to be minimal memory effects. Nevertheless, post hoc, we hypothesized that the greater the delay between the training and when the rater next assesses the participants, the more likely they would revert to the particular approach for applying the scale they used in Round 1 before the training. Although this trend was evident for all of the scales, it was not significant. In any case, the time interval between training and subsequent ratings should be systematically examined further in future studies using training.

### Recommendations

At least in our application context, consensus training did not significantly improve IRR. This suggests that the training process could be omitted without a significant reduction in IRR. Because consensus training requires coordinating one or more meetings among multiple busy clinicians, omitting the training could dramatically improve efficiency and lower the cost of future studies using these HFS scales.

In terms of specific HFS scales, the best choice for future HFS motor severity assessments should depend on the application. For HFS research studies seeking more detailed quantification of different HFS motor manifestations, the correspondingly more detailed scales may be appropriate. In clinical settings including trials, based on the results of our study, Tunc’s scale should be prioritized for multiple reasons. First, it exhibited the highest reliability. Second, its reliability seemed to depend least on consensus training and is therefore robust. Third, like clinical global impressions of severity, it has the simplest wording. This makes the scale easy to interpret without the need for any modification or training, and therefore practical to use across multiple raters.

### Limitations

Our study has several limitations. First, our results are specific to brief, video-based, observational assessments and may not translate to longer, in-person, interactive use. Notably, activation procedures are explicitly accommodated in some variations of the scales. For example, at least some versions of the AIMS (Guy 1976) on which Tunc’s scale is based include instructions to subtract one point “if movements are seen only in activation.” Therefore, future studies should consider including procedures that evoke the participant’s symptoms, ensure that the procedures are implemented consistently, and consider associated modified ratings when assessing the reliability of HFS rating scales. Second, because our focus was to simplify the language of the scales and observe the resulting IRR after operationalizing these revisions, none of the clinical rating scales in this study were revised to include specific separate evaluation of the cheeks. Although altering one or more of the scales to incorporate separate cheek evaluation would enable more detailed assessment, it could also complicate rater interpretation and decrease IRR. Third, we cannot exclude the possibility that some raters’ ratings were biased by having seen some of the patients before. Some of the raters saw a small subset of the patients during initial video recordings as part of a separate study. However, that study was completed over 1 year prior to the start of this study, and most patients were previously unseen by each rater. Fourth, we cannot entirely exclude the possibility of a learning effect; raters’ assessments in Round 2 may have been biased by Round 1 assessments. However, this should have been minimal, not only because they were explicitly instructed to ignore their Round 1 ratings, but also because there was at least a 138-day delay between the two rounds of rating. Fifth, the raters were not given explicit instructions about the order in which to use the scales. However, the document including the scales and the spreadsheet in which they noted their ratings had all of the scales in the same order. Thus, we cannot exclude the possibility of an order effect, i.e., the order in which the scales were used influencing their relative reliability. However, we deem this effect to be minimal given the dramatic differences in how the scales are worded. Sixth, the scale adaptations and consensus training were conducted as a unitary process, so their individual influences on how IRR improved from Round 1 to Round 2 cannot be disentangled. Finally, we did not evaluate intra-rater reliability, which would be useful information in contexts of repeated assessments by the same rater. Strictly speaking, an evaluation of intra-rater reliability should not include any changes in the rating instrument between the two instances of ratings. In this study, how the scales were interpreted and operationalized differed between the two instances. This would confound interpretation of any intra-rater differences between the two instances.

## Conclusion

Because video-based assessments are playing an expanding role in multisite trials, telehealth, and efforts to increase accessibility to expert care, it is becoming increasingly important to optimize the reliability of video-based measures of motor severity in HFS. Our results point to the use of a simplified scale as the preferred option to (1) facilitate clinician-based severity ratings, (2) provide an anchor against which to validate emerging objective, computational methods for quantifying HFS motor symptoms (Peterson et al. 2016; Osaki et al. 2020a), and (3) complement advances in patient-centered instruments for measuring the combined impact of both motor and non-motor aspects of HFS on quality of life (Wabbels and Yaqubi 2021).

## Data Availability

Data available from the Dystonia Coalition upon reasonable request.

## Appendix A: HFS clinical rating scales

NOTES:

1. Use only the “Observation” period (for ALL patients and ALL scales)
2. Wording greyed out with strikethrough should be DISREGARDED.
3. “Fluttering” (JRS, Chen) defined as: blinks occurring in rapid succession, sometimes without complete opening of the eye between blinks (per Jinnah’s Blepharospasm Phenotyping Tool, under development)

## Appendix B: Inter-round rating consistency

**Table:**

For each scale, the percentage of ratings that differed between Rounds 1 and 2 by these many points:			
Scale	0	1	> 1
JRS	54	23	23
HSGS	51	13	36
Lee	73	16	11
Chen	61	20	19
Tunc	65	32	3

## Appendix D: Proportion of raters being within one point of each other

**Table:**

For each scale, the proportion of ratings for which the raters were within one point of each other (%):		
Scale	Round 1	Round 2
JRS	2	16
HSGS	7	16
Lee	40	44
Chen	16	31
Tunc	60	53
